# RONIN/HCF1‐TFEB Axis Protects Against D‐Galactose‐Induced Cochlear Hair Cell Senescence Through Autophagy Activation

**DOI:** 10.1002/advs.202407880

**Published:** 2025-02-22

**Authors:** Yongjie Wei, Yuhua Zhang, Wei Cao, Nan Cheng, Yun Xiao, Yongjun Zhu, Yan Xu, Lei Zhang, Lingna Guo, Jun Song, Su‐hua Sha, Buwei Shao, Fang Ma, Jingwen Yang, Zheng Ying, Zuhong He, Renjie Chai, Qiaojun Fang, Jianming Yang

**Affiliations:** ^1^ Department of Otolaryngology‐Head and Neck Surgery The Second Affiliated Hospital of Anhui Medical University Hefei 230601 China; ^2^ Department of Pathology and Laboratory Medicine The Medical University of South Carolina Charleston SC 29425 USA; ^3^ School of Medicine Faculty of Medical & Health Sciences Tel Aviv University Tel Aviv 6997801 Israel; ^4^ Center for Scientific Research of Anhui Medical University Hefei 230032 China; ^5^ International Department of Hefei 168 High School Hefei 230601 China; ^6^ Jiangsu Key Laboratory of Neuropsychiatric Diseases and College of Pharmaceutical Sciences Soochow University Suzhou 215123 China; ^7^ Department of Otorhinolaryngology‐Head and Neck Surgery Zhongnan Hospital of Wuhan University Wuhan 430071 China; ^8^ State Key Laboratory of Digital Medical Engineering Department of Otolaryngology Head and Neck Surgery Zhongda Hospital School of Life Sciences and Technology School of Medicine Advanced Institute for Life and Health Jiangsu Province High‐Tech Key Laboratory for Bio‐Medical Research Southeast University Nanjing 210096 China; ^9^ Co‐Innovation Center of Neuroregeneration Nantong University Nantong 226001 China; ^10^ Department of Neurology Aerospace Center Hospital School of Life Science Beijing Institute of Technology Beijing 100081 China; ^11^ Department of Otolaryngology Head and Neck Surgery Sichuan Provincial People's Hospital University of Electronic Science and Technology of China Chengdu 610072 China; ^12^ Southeast University Shenzhen Research Institute Shenzhen 518063 China

**Keywords:** aging‐related hearing loss, autophagy, hair cell, RONIN/THAP11, Tfeb

## Abstract

Age‐related hearing loss is characterized by senescent inner ear hair cells (HCs) and reduced autophagy. Despite the improved understanding of these processes, detailed molecular mechanisms underlying cochlear HC senescence remain unclear. Transcription Factor EB (TFEB), a key regulator of genes associated with autophagy and lysosomes, crucially affects aging‐related illnesses. However, intricate regulatory networks that influence TFEB activity remain to be thoroughly elucidated. The findings revealed that RONIN (THAP11), through its interaction with host cell factor C1 (HCF1/HCFC1), modulated the transcriptional activity of *Tfeb*, thus contributing to the mitigation (D‐galatactose [D‐gal]) senescent HC loss. Specifically, RONIN overexpression improved autophagy levels and lysosomal activity and attenuated changes associated with the senescence of HCs triggered by D‐gal. These findings highlight the possibility of using RONIN as a viable therapeutic target to ameliorate presbycusis by enhancing the TFEB function.

## Graphical abstract

D‐galactose (D‐gal) induced inner ear hair cell senescence by inhibiting TFEB transcription. RONIN/HCF1 promotes TFEB transcription to prevent cochlear HCs from D‐gal‐induced senescence through autophagy activation.

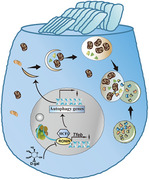

## 1 Introduction

Age‐related hearing loss (ARHL) or presbycusis is a progressive, irreversible auditory dysfunction resulting from cochlear aging. Roughly 66% of adults aged 70 years and above suffer from hearing impairment.^[^ ^1^ ^]^ Globally, the population of individuals aged over 60 years exceeds 1.2 billion, with an estimated 500 million anticipated suffering from ARHL.^[^ ^2^ ^]^ ARHL is an intricate and multifaceted condition characterized by a decline in auditory abilities. Several variables, such as the environment, genetics, overall health, and diet, affect ARHL.^[^ ^3^ ^]^ Despite the existing knowledge, mechanisms regulating age‐related cochlear degeneration at the cellular and molecular levels remain poorly understood. Consequently, investigating these mechanisms is necessary to identify interventions that can delay the onset of HC senescence.

Autophagy, or macroautophagy, is a universal intracellular mechanism that efficiently breaks down and recycles metabolic waste in all eukaryotic organisms. This process removes abnormal proteins, impaired organelles, and other harmful substances from cells to enhance their viability.^[^ ^4^, ^5^ ^]^ Mounting evidence suggests that aging is linked to a gradual deterioration in autophagic activity.^[^ ^6^, ^7^ ^]^ Moreover, mutations in critical autophagy genes can affect lifespan, and autophagy dysfunction is linked to the onset of various neurodegenerative diseases.^[^ ^8^ ^]^ Autophagy plays a crucial role in HC injury. He et al. demonstrated that aminoglycoside‐induced damage elevates autophagy levels in HCs. Autophagy agonists enhance HC survival and inhibition of autophagy intensifies apoptosis in these cells.^[^ ^9^ ^]^ Autophagy activation offers protection against cisplatin‐induced damage to spiral neurons.^[^ ^10^ ^]^ Autophagy levels decrease in the cochlear HCs of aged deaf mice; Enhanced autophagy reduces HC loss and improves hearing.^[^ ^11^ ^]^ However, further studies are required to elucidate the regulatory mechanisms underlying autophagy in ARHL.

Transcription Factor EB (TFEB) belongs to the microphthalmia‐associated transcription factor family, specifically to the class of basic helix‐loop‐helix leucine zipper (bHLH‐LZ) proteins. It is a downstream target of the mTOR pathway and regulates gene expression through nucleation. TFEB plays pivotal roles in autophagy and lysosomal biogenesis.^[^ ^12^ ^]^ Diminished nuclear localization of TFEB is linked to the progression of various neurodegenerative diseases.^[^ ^13^, ^14^, ^15^ ^]^ Enhancing TFEB expression and nuclear localization using small‐molecule drugs can improve autophagy, thereby reducing damage to HCs and spiral neurons.^[^ ^16^, ^17^ ^]^ Nevertheless, the precise mechanisms by which TFEB controls autophagy in ARHL remain unclear. We hypothesize that modulating TFEB expression and autophagy can offer a novel approach to treating age‐related deafness.

In this study, we utilized previously reported D‐gal‐induced cellular and tissue models to unveil the roles of TFEB and autophagy in ARHL.^[^ ^18^, ^19^, ^20^ ^]^ We found that RONIN enhanced TFEB transcriptional activity by interacting with HCF1. Elevated *Ronin* levels activated autophagy and promoted the survival of cochlear HCs. Based on these observations, targeting the RONIN/HCF1‐TFEB pathway may offer a novel therapeutic strategy for preventing HC degeneration in ARHL.

## 2 Results

### 2.1 D‐Gal Induced Senescence in HEI‐OC1 Cells and Cochlear Explants

**Figure 1:**
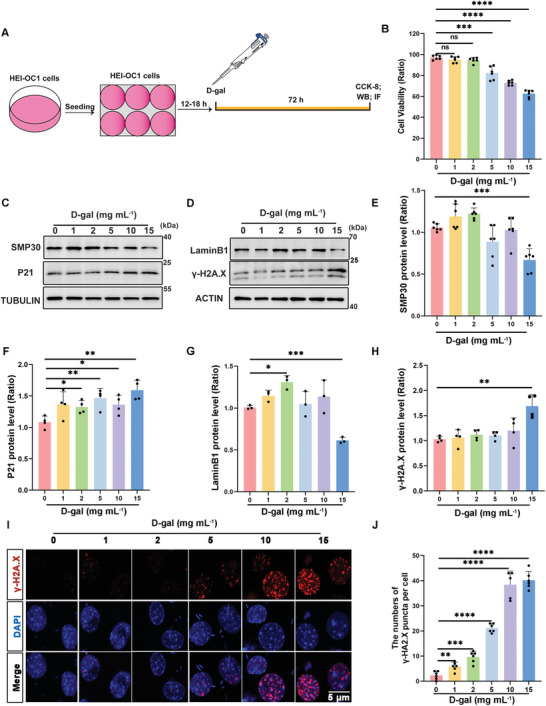
HEI‐OC1 cells were subjected to senescence induction by treatment with D‐gal. A) HEI‐OC1 cells following treatment with D‐gal. B) The results of the CCK‐8 assay demonstrate the survival of cells following treatment with different doses of D‐gal for a duration of 72 h, n = 6. Error bars are ± S. D., ns: no significant difference, ^***^ *p* < 0.001 and ^****^ *p* < 0.0001. C,D) Western blot analysis to identify senescence markers post 72 h treatment with different D‐gal concentrations in HEI‐OC1 cells. E–H) Quantitative examination of the western blot data, n = 6 for E, n = 4 for F, n = 3 for G, n = 4 for H. Error bars are ± S.D., ^*^ *p* < 0.05, ^**^ *p* < 0.01 and ^***^ *p* < 0.001. I) Immunofluorescence staining depicting the distribution of γ‐H2A.X foci in the nuclei (stained with DAPI) of HEI‐OC1 cells treated with D‐gal for 72 h, Scale bar: 5 µm. J) Quantitative analysis of γ‐H2A.X foci in the nuclei, n = 6. Error bars are ± S.D., ^**^ *p* < 0.01, ^***^ *p* < 0.001, and ^****^ *p* < 0.0001.

Natural aging is an extended biological process. Since its introduction in 1962, D‐gal has been widely used in aging research.^[^ ^21^, ^22^ ^]^ HEI‐OC1 cells, a line closely resembling HC and exhibiting HC properties, have been extensively used to examine the biological processes of HC damage and preservation.^[^ ^23^, ^24^ ^]^ In our experiments to delineate aging‐induced HC damage, we induced senescence in HEI‐OC1 cells using varying concentrations of D‐gal over 72 h (**Figure** **1**A). Cell viability assessments using the Cell Counting Kit‐8 (CCK‐8) reagent revealed a notable decline in cell survival at concentrations exceeding 5 mg mL^−1^ (Figure 1B). Additionally, western blot analyses demonstrated changes in levels of senescence‐related markers (SMP30, P21, LaminB1, and γ‐H2A.X) (Figure 1C, D),^[^ ^25^, ^26^, ^27^, ^28^ ^]^ with the expression of P21 and γ‐H2A.X increasing significantly at 15 mg mL^−1^ D‐gal treatment; the expressions of SMP30 and LaminB1 proteins decreased (Figure 1E–H). These results suggested that 15 mg mL^−1^ D‐gal triggered senescence in HEI‐OC1 cells. Subsequent immunofluorescence staining for γ‐H2A.X demonstrated an increase in nuclear spots post‐treatment (Figure 1I,J).

The senescence‐associated β‐galactosidase (SA‐β‐gal) cell senescence detection assay was employed for the precise identification and quantification of cellular senescence. Following D‐gal treatment, SA‐β‐Gal staining showed a significantly higher integrated optical density (Figure S1A, B, Supporting Information). These results provide evidence of effective induction of D‐gal‐induced senescence in HEI‐OC1 cells.

**Figure 2:**
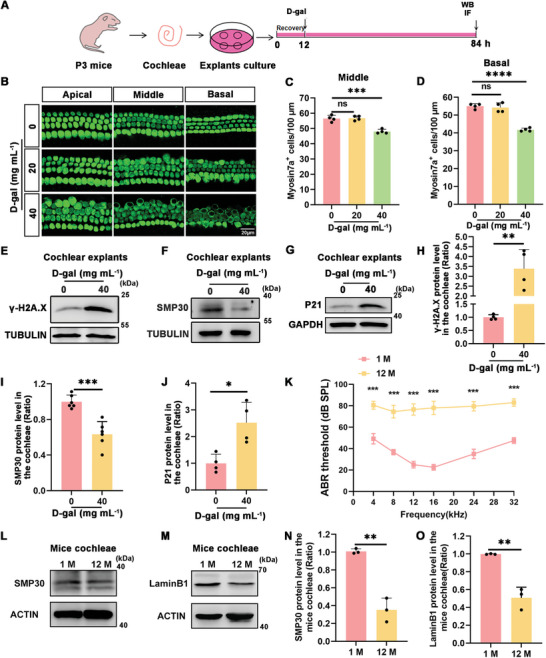
Senescence in cochlear explants treated with D‐gal and aging mice. A) Cochleae cultured in vitro and treated with D‐gal. B) Immunofluorescence staining for Myosin7a, demonstrating cochlear HC loss after 72 h of D‐gal treatment (20 and 40 mg mL^−1^), Scale bar: 20 µm. C,D) Measurement of cell numbers expressing Myosin7a in the middle and basal turns of cochlea, n = 4. Error bars are ± S.D., ns: no significant difference, ^***^ *p* < 0.001 and ^****^ *p* < 0.0001. E–G) Western blot results showing the expression of senescence‐related markers (γ‐H2A.X, SMP30, and P21) in cochleae following 72 h of treatment with D‐gal. H‐J) Quantitative analysis of western blot data, n = 4 for H, n=6 for I, n=4 for J. Error bars are ± S.D., ^*^ *p* < 0.05, ^**^ *p* < 0.01, ^***^ *p* < 0.001. K) ABR threshold in 1 M (1 month) and 12 M (12 months) C57BL/6J mice. n = 3. Error bars are ± S.D., ^***^ *p* < 0.001. L,M) The results of western blots show the expression of senescence‐related markers in cochleae of aged mice. N,O) Quantification of SMP30 and LaminB1 protein levels using western blot, n = 3. Error bars are ± S.D., ^**^ *p* < 0.01.

To establish a D‐gal‐induced aging‐mimetic tissue model, we dissected cochlear basilar membranes from 3‐day postnatal (P3) mice for in vitro culture. These samples were subjected to 20 and 40 mg mL^−1^ D‐gal treatment for 72 h (**Figure** **2**A). Immunofluorescence staining and subsequent HC counting revealed no HC loss following treatment with 20 mg mL^−1^ D‐gal (Figure 2B–D). In contrast, treatment with 40 mg mL^−1^ D‐gal significantly reduced HCs in the middle and basal region of the cochlea (Figure 2B–D). Western blot analysis of the cochleae treated with 40 mg mL^−1^ D‐gal for 72 h showed elevated levels of γ‐H2A.X and P21 and decreased SMP30 expression (Figure 2E–J). These results suggested that exposure to 40 mg mL^−1^ D‐gal for 72 h was sufficient to induce HC senescence. Auditory thresholds and HC senescence were detected in 1‐ (1 M) and 12‐month (12 M) mice. The results showed impaired auditory function and decreased expression of senescence markers, SMP30 and LaminB1 (Figure 2K–O), suggesting ARHL in 12 M mice. Thus, this model is effective for studying the effects of aging on cochlear cells and can help guide future research on therapeutic interventions for ARHL.

### 2.2 Autophagy Levels Decreased in Senescent HEI‐OC1 Cells, Cochlear Explants, and Cochleae of Aged Mice

**Figure 3:**
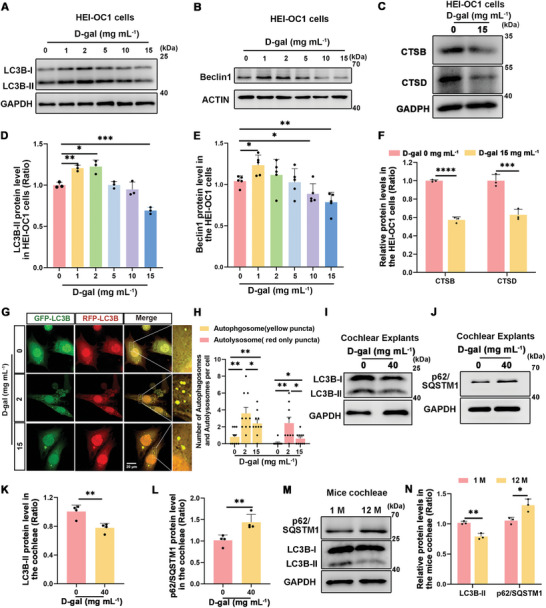
Autophagy is impaired in HEI‐OC1 cells and cochlear explants after treatment with D‐gal and in aged mice cochleae. A) LC3B expression in HEI‐OC1 cells was measured by western blot analysis. HEI‐OC1 cells were treated with different concentrations of D‐gal for 72 h. B) Western blot showed Beclin1 expression in HEI‐OC1 cells. HEI‐OC1 cells were treated with different concentrations of D‐gal for 72 h. C) Western blots demonstrate altered CTSB and CTSD protein concentrations in HEI‐OC1 cells following exposure to 15 mg mL^−1^ D‐gal treatment for 72 h. D) Quantification of LC3B‐II protein levels in HEI‐OC1 cells in A, Error bars are ± S.D., n = 3, ^*^ *p* < 0.05, ^**^ *p* < 0.01, ^***^ *p* < 0.001. E) Quantification of Beclin1 protein levels in HEI‐OC1 cells in B, n = 5. Error bars are ± S.D., ^*^ *p* < 0.05 and ^**^ *p* < 0.01 F) Quantification of CTSB and CTSD protein levels in HEI‐OC1 cells in C, n = 3, Error bars are ± S.D., ^***^ *p* < 0.001 and ^****^ *p* < 0.0001. G) Images of RFP‐GFP‐LC3B puncta in HEI‐OC1 cells. The cells were transfected with RFP‐GFP‐LC3B plasmids and exposed to 2 and 15 mg mL^−1^ D‐gal for 72 h, Scale bar: 20 µm. Yellow and red dots represent autophagosomes and autolysosomes, respectively. H) Quantitative assessment of autophagosomes and autolysosomes, n = 10. Error bars are ± S.D., ^*^ *p* < 0.05 and ^**^ *p* < 0.01. I,J) Western blot results showing changes in LC3B and p62/SQSTM1 expression in cochlear explants treated with 40 mg mL^−1^ D‐gal for 72 h. K,L) Quantification of LC3B‐II and p62/SQSTM1 protein levels in I and J, n = 4. Error bars are ± S.D., ^**^ *p* < 0.01. M) Western blot results showing changes in LC3B and p62/SQSTM1 expression in aged mice cochleae. N) Quantification of LC3B‐II and p62/SQSTM1 protein levels in M, n = 3. Error bars are ± S.D., ^*^ *p* < 0.05 and ^**^ *p* < 0.01.

We first analyzed the expression of autophagy markers (LC3B, Beclin1, CTSB, CTSD, p62/SQSTM1, and ATG7) following treatment with varying D‐gal concentrations to assess autophagy levels in senescent HEI‐OC1. Western blot analysis revealed increased expression of LC3B‐II and Beclin1 at lower concentrations (1‐2 mg mL^−1^), suggesting cytoprotective autophagy (**Figure** **3**A, B, D, and E). In contrast, higher concentrations (above 5 mg mL^−1^) led to a decrease in autophagy levels, suggesting the disruption of mechanisms of cellular autophagy following D‐gal treatment. The accumulation of p62/SQSTM1 and reduction in ATG7 protein expression suggested that autophagy levels were progressively suppressed with increasing D‐gal concentrations (Figure S2A, B, E, and F, Supporting Information). Additionally, western blotting revealed decreased expression of lysosomal hydrolases, CTSB and CTSD, following D‐gal treatment (Figure 3C, F), corroborating lysosomal dysfunction. Furthermore, we used RFP‐GFP‐LC3B plasmids to measure the number of autophagosomes and autolysosomes.^[^ ^29^ ^]^ The findings revealed a noteworthy increase in both yellow (autophagosomes) and red spots (autolysosomes) in cells exposed to lower concentrations of D‐gal compared to the control group. There was a notable reduction in the levels of these markers at 15 mg mL^−1^ D‐gal, indicative of impaired autophagic flux (Figure 3G, H). These findings align with those of recent studies indicating lysosomal dysfunction as a primary factor in autophagy deficits observed in various neurodegenerative diseases.^[^ ^30^, ^31^ ^]^ Consistent with the results of our previous study, 2 mg mL^−1^ D‐gal treatment activated autophagy, whereas 15 mg mL^−1^ D‐gal treatment significantly suppressed autophagy in senescent HEI‐OC1 cells. For subsequent experiments, we used 2 mg mL^−1^ D‐gal to detect autophagy activation and 15 mg mL^−1^ D‐gal to test cellular senescence.

Western blot analysis demonstrated a reduction in LC3B‐II protein levels (Figure 3I, K) and an increase in the level of the p62/SQSTM1 protein (Figure 3J, L) in D‐gal‐treated aged cochlear explants. Changes in LC3B‐II and p62/SQSTM1 protein levels in aged mice cochleae were consistent with those in D‐gal‐treated aged cochlear explants (Figure 3M, N), and Beclin1, and ATG7 protein levels also decreased in cochleae of aged mice (Figure S2C, D, G, and H, Supporting Information). These findings indicate that autophagy is suppressed in the cochlea, consistent with the results reported for HEI‐OC1 cells.

### 2.3 Increased Accumulation of Damaged Mitochondria in Senescent HEI‐OC1 Cells

**Figure 4:**
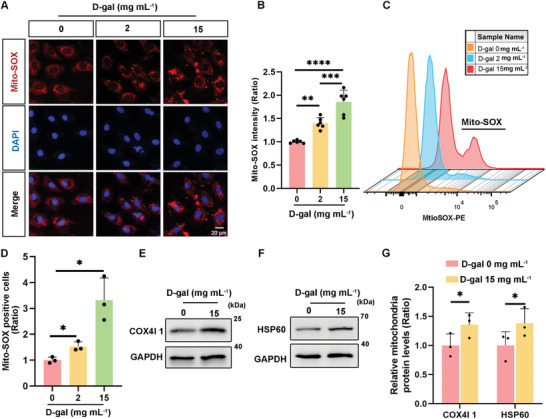
Increase in damaged mitochondria in senescent HEI‐OC1 cells induced by D‐gal treatment. A) Mito‐SOX staining results indicate increased levels of oxidative stress in cells after 72 h of exposure to varying concentrations of D‐gal, Scale bar: 20 µm. B) Analysis of Mito‐SOX fluorescence intensity within cells, n = 6. Error bars are ± S.D., ^**^ *p* < 0.01, ^***^ *p* < 0.001, and ^****^ *p* < 0.0001. C) Flow cytometry analysis of ROS levels in HEI‐OC1 cells using Mito‐SOX. HEI‐OC1 cells treated with 2 and 15 mg mL^−1^ D‐gal for 72 h. D) Quantification of ROS levels in C. n = 3. Error bars are ± S.D., ^*^ *p* < 0.05. E,F) Western blot analysis shows changes in the levels of mitochondrial marker proteins in HEI‐OC1 cells following 72 h of treatment with 15 mg mL^−1^ D‐gal. G) Quantitative analysis of western blot results in E and F, n = 3. Error bars are ± S.D., ^*^ *p* < 0.05.

To examine the relationship between mitochondrial impairment and the process of aging, we assessed how mitochondrial malfunction, a known driver of reactive oxygen species (ROS) accumulation, contributed to markers of aging and apoptosis.^[^ ^32^, ^33^ ^]^ We employed Mito‐SOX Red staining and flow cytometry to measure the levels of ROS in aged HEI‐OC1 cells. Intracellular ROS levels significantly increased after D‐gal treatment (**Figure** **4**A–D), suggesting that D‐gal‐induced mitochondrial damage. The methodology was based on a prior study that showed the correlation of mitochondrial distress with cellular aging.^[^ ^32^ ^]^ Further analysis by western blotting revealed significant increases in the expression of mitochondrial inner membrane proteins (COX4I1) and mitochondrial matrix proteins (HSP60) (Figure 4E, F, and G), confirming the accumulation of damaged mitochondria in these cells.

### 2.4 TFEB Expression Decreased in D‐Gal‐Induced Senescent HEI‐OC1 Cells, Cochlear Explants, and Cochleae of Aged Mice

**Figure 5:**
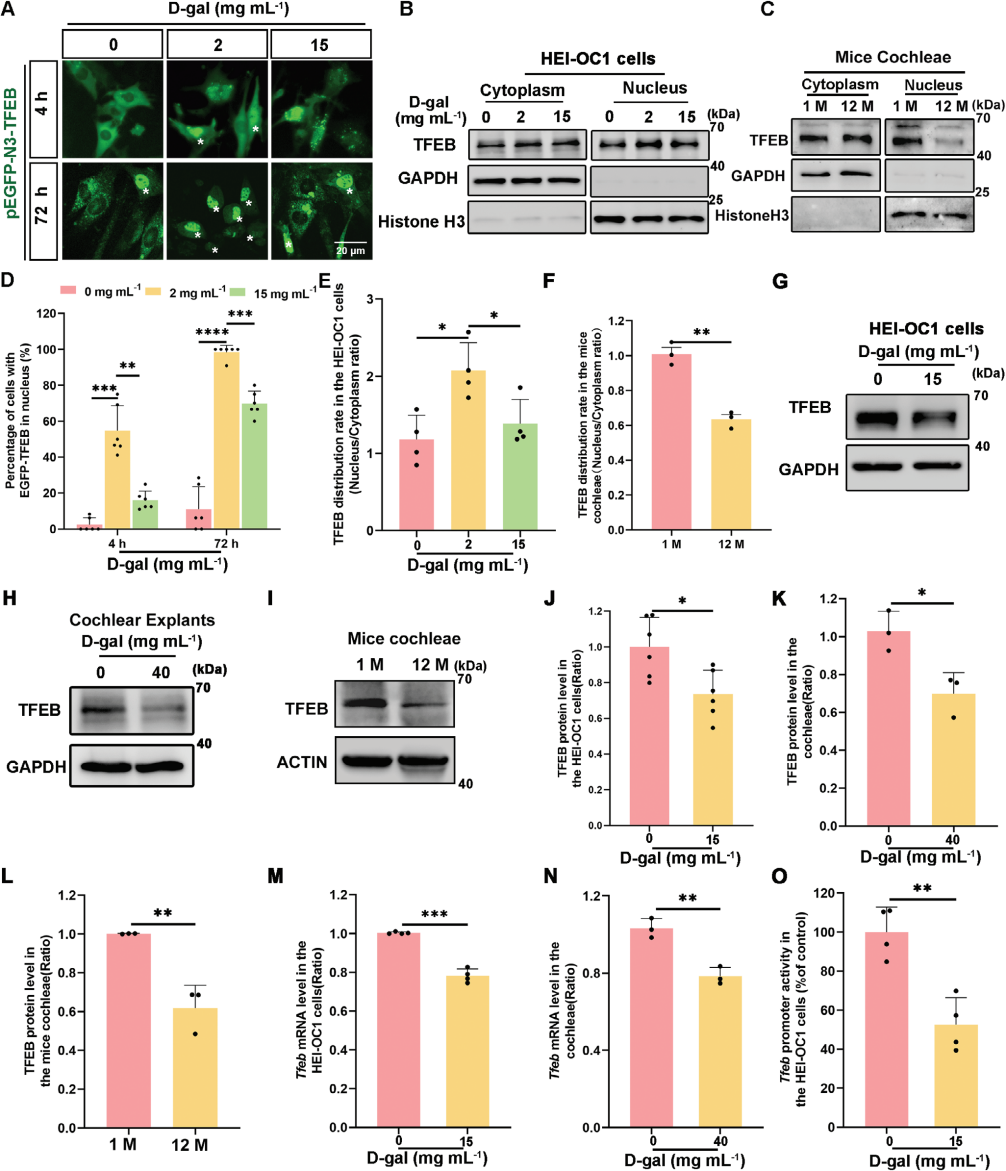
Suppression of TFEB transcriptional activity in HEI‐OC1 cells and cochleae following D‐gal treatment. A) Visualization of TFEB distribution within the nucleus of HEI‐OC1 cells under different treatment conditions, after transfection with pEGFP‐N3‐TFEB, Scale bar: 20 µm. B) Western blot analysis detecting changes in TFEB expression within the nucleus and cytoplasm of HEI‐OC1 cells after 72 h of D‐gal treatment. C) Western blot showing the changes in TFEB distribution in the cytoplasm and nucleus between 1 M and 12 M mice cochleae. D) Quantitative analysis of cells exhibiting nuclear entry of TFEB, n = 6. Error bars are ± S.D., ^**^ *p* < 0.01, ^***^ *p* < 0.001, and ^****^ *p* < 0.0001. E) Quantitative analysis of western blot results about TFEB distribution in B, n = 4. Error bars are ± S.D., ^*^ *p* < 0.05. F) Quantification of TFEB distribution rate in cytoplasm and nuclear fractions in C, n = 3. Error bars are ± S.D., ^**^ *p* < 0.01. G) Western blot analysis of TFEB protein expression in HEI‐OC1 cells after 72 h of D‐gal treatment. H) Western blot analysis showing changes in TFEB expression in cochlear explants after D‐gal treatment for 72 h. I) Western blot showing the changes in TFEB expression between 1 M and 12 M mice cochleae. J–L) Quantitative analysis of TFEB protein levels, n = 6 for J, n = 3 for K, n = 3 for L. Error bars are ± S.D., ^*^ *p* < 0.05 and ^**^ *p* < 0.01. M,N) qPCR analysis for assessing changes in *Tfeb* mRNA level in HEI‐OC1 cells and cochlear explants, n = 4 for M, n = 3 for N. Error bars are ± S.D., ^**^ *p* < 0.01 and ^***^ *p* < 0.001. O) *Tfeb* luciferase activity assay demonstrating a significant decrease in TFEB transcriptional activity after treatment with 15 mg mL^−1^ D‐gal, n = 4. Error bars are ± S.D., ^**^ *p* < 0.01.

TFEB is essential for maintaining lysosomal formation and controlling autophagy.^[^ ^12^, ^34^ ^]^ Our results indicate that lysosomal hydrolases and autophagy activity are suppressed in senescent HEI‐OC1 cells, likely due to the disruption in TFEB signaling pathways. TFEB, a nucleoplasm shuttle protein, regulates gene transcription in the nucleus. We treated HEI‐OC1 cells expressing EGFP‐TFEB with 2 and 15 mg mL^−1^ D‐gal for 72 h to ascertain the effect of D‐gal on TFEB nuclear translocation. Imaging studies revealed that 2 mg mL^−1^ D‐gal promoted nuclear translocation of TFEB, further promoting autophagy. Compared with 2 mg mL^−1^ D‐gal, the number of HEI‐OC1 cells exhibiting TFEB nuclear translocation reduced significantly after 15 mg mL^−1^ D‐gal treatment (**Figure** **5**A, D). These findings were consistent with autophagy repression in HEI‐OC1 cells following treatment with 15 mg mL^−1^ D‐gal. Thus, HEI‐OC1 cells were treated with 15 mg mL^−1^ D‐gal in subsequent experiments to induce cellular senescence. Nucleus‐cytoplasm fractionation experiments confirmed reduced nuclear translocation of TFEB at higher D‐gal concentrations (Figure 5B, E). Nuclear TFEB distribution was decreased in the cochlea of aged mice, indicating that D‐gal‐induced senescence was similar to natural aging (Figure 5C, F). Furthermore, a notable decrease in total TFEB protein levels was observed in D‐gal‐treated HEI‐OC1 cells, cochlear explants, and naturally aging mice (Figure 5G–L), suggesting the inhibition of TFEB expression after cell senescence. Additionally, *Tfeb* mRNA levels decreased significantly after HEI‐OC1 cells and cochlear tissues were exposed to D‐gal (Figure 5M, N), implying that D‐gal impaired autophagy by inhibiting the transcriptional activity of *Tfeb*. *Tfeb* dual‐luciferase reporter assay further demonstrated that D‐gal repressed *Tfeb* promoter activity in aged HEI‐OC1 cells (Figure 5O).

### 2.5 The Combination of RONIN with HCF1 Enhances TFEB Transcription

**Figure 6:**
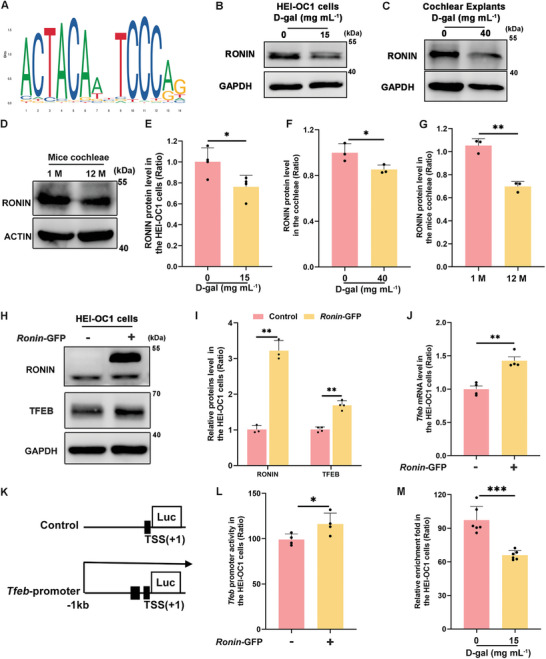
Decreased expression of *Ronin* inhibits TFEB transcription in senescent HEI‐OC1 Cells. A) The RONIN‐binding motif (ACTACNNTCCCAG) was predicted using the JASPAR database, and schematic images of the potential RONIN binding sites in the *Tfeb* promoter region are shown. B–D) Western blot analysis was conducted to measure the amounts of RONIN protein in aged HEI‐OC1 cells, cochlear explants, and aged mice cochleae. HEI‐OC1 cells were treated with 15 mg mL^−1^ D‐gal for 72 h, and cochlear explants were treated with 40 mg mL^−1^ D‐gal for 72 h. E–G) Statistical analysis of western blot results of RONIN expression in HEI‐OC1 cells, cochlear explants, and mice cochleae. n = 4 for E, n = 3 for F, n = 3 for G. Error bars are ± S.D., ^*^ *p* < 0.05 and ^**^ *p* < 0.01. H) Western blot analysis of RONIN and TFEB protein levels in HEI‐OC1 cells following overexpression of *Roni*n by transfecting with the *Ronin*‐GFP plasmid for 48 h. I) Statistical evaluation of RONIN and TFEB protein levels, n = 3 for RONIN analysis; n = 4 for TFEB analysis. Error bars are ± S.D., ^**^ *p* < 0.01. J) qPCR analysis of *Tfeb* mRNA expression in HEI‐OC1 cells transfected with the *Ronin*‐GFP plasmids, n = 4. Error bars are ± S.D., ^**^ *p* < 0.01. K) The diagram illustrates the *Tfeb* luciferase promoter‐reporter construct and the direction of transcription initiation is shown by an arrow in the figure. L) Assessment of *Tfeb* promoter activity following *Ronin* overexpression in transfected cells, n = 4. Error bars are ± S.D., ^*^ *p* < 0.05. M) Results of the CUT&RUN‐qPCR assay show reduced relative fold enrichment of *Tfeb* promoter from DNA precipitated by anti‐RONIN antibody in HEI‐OC1 cells after 15 mg mL^−1^ D‐gal treatment. Cells were transfected with *Ronin*‐GFP, n = 6. Error bars are ± S.D., ^***^ *p* < 0.001.

We explored the potential transcription factors using the JASPAR database to elucidate the mechanisms underlying the decline in *Tfeb* transcription levels. We identified RONIN, a member of the Thanatos‐associated protein (THAP) family characterized by a zinc‐dependent, sequence‐specific DNA‐binding domain at its N‐terminus, which recognizes the DNA sequence ACTACNNTCCCAG and belongs to the zinc‐finger superfamily (**Figure** **6**A).^[^ ^35^ ^]^ Given that RONIN, a murine homolog of the human THAP11 gene, is highly conserved and can regulate genes crucial for mitochondrial function and cell survival,^[^ ^36^ ^]^ we hypothesized that RONIN could mitigate cellular senescence by modulating TFEB transcription. Western blot analysis showed significantly decreased RONIN protein levels in HEI‐OC1 cells and cochlear explants post 72 h D‐gal treatment and in naturally aged mice cochlea (Figure 6B–G; Figure S3, Supporting Information). Cells expressing *Ronin*‐GFP exhibited a marked increase in TFEB protein and *Tfeb* mRNA levels (Figure 6H–J). The *Tfeb* dual‐luciferase reporter assay indicated enhanced *Tfeb* promoter activity following *Ronin* overexpression (Figure 6K, L). A CUT and RUN qPCR assay^[^ ^37^ ^]^ was performed, which demonstrated that RONIN regulated TFEB promoter activity (Figure 6M). Our findings suggest that RONIN enhance*s Tfeb* transcription by binding to a region within 1000 bp upstream of the *Tfeb* promoter in HEI‐OC1 cells. HCF1 is a transcriptional co‐regulator with fundamental biological functions, including transcriptional control and cell cycle progression.^[^ ^38^, ^39^, ^40^ ^]^ Because HCF1 and RONIN interact to control the transcription of certain genes, our objective was to examine the function and mechanism of HCF1 in facilitating TFEB transcription through RONIN.^[^ ^41^ ^]^ Our results showed that HCF1 could bind to RONIN in HEI‐OC1 cells (Figure S4A, Supporting Information). Interestingly, the results of western blot analysis showed no changes in HCF1 protein levels, either following D‐gal treatment alone or by D‐gal treatment after RONIN overexpression in HEI‐OC1 cells (Figure S4B–E, Supporting Information). However, *Tfeb* mRNA levels were considerably reduced after *Hcf1* siRNA treatment compared to the group with *Ronin* overexpression (Figure S4F–H, Supporting Information). These data indicate that HCF1 participates in RONIN by promoting the transcription of *Tfeb*.

### 2.6 Activation of Ronin in Cochlear HCs Protects Against Senescence

**Figure 7:**
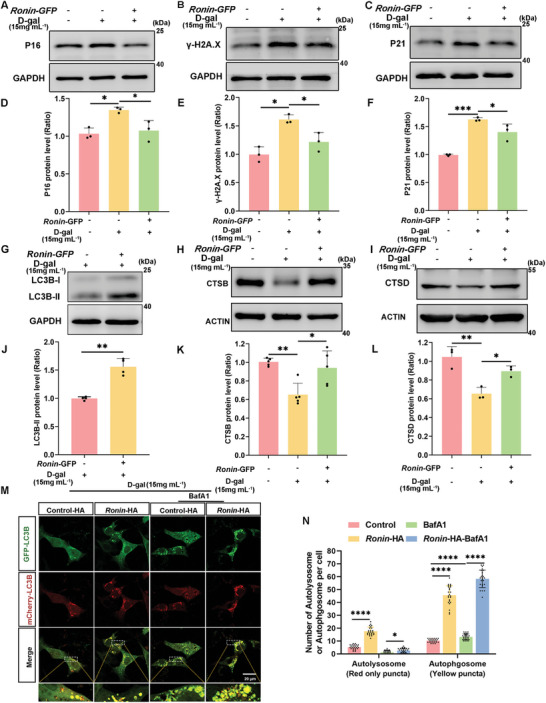
Activation of autophagy by *Ronin* overexpression rescues cellular senescence. A–C) Western blot examination shows alterations in the protein levels of P16, γ‐H2AX, and P21 in HEI‐OC1 cells treated with 15 mg mL^−1^ for 72 h D‐gal after transfection with *Ronin*‐GFP plasmids. D–F) Densitometric quantification of the western blot bands for protein as indicated in (A–C), n = 3. Error bars are ± S.D., ^*^ *p* < 0.05 and ^***^ *p* < 0.001. G–I) Western blots illustrating alterations in the levels of autophagy markers LC3B‐II, CTSB, and CTSD in HEI‐OC1 cells treated with 15 mg mL^−1^ D‐gal post‐transfection with *Ronin*‐GFP. J–L) Quantification of LC3B‐II, CTSB, and CTSD expression, n = 4 for J, n = 5 for K, n = 3 for L. Error bars are ± S.D., ^*^ *p* < 0.05, ^**^ *p* < 0.01. M) Images of mCherry‐GFP‐LC3B punctae in HEI‐OC1 cells. Cells were transfected with pcsLenti‐CMV‐mCherry‐GFP‐LC3B (Lentivirus) and *Ronin*‐HA plasmids, followed by exposure to 15 mg mL^−1^ D‐gal for 72 h with or without 100 nM bafliomycinA1 (BafA1) for 6 h. Autophagosomes and autolysosomes are represented by yellow and red dots, respectively. N) Quantification of mCherry‐GFP‐LC3B yellow and red punctae in M, n = 24. Error bars are ± S.D., ^*^ *p* < 0.05 and ^****^ *p* < 0.0001.

*Ronin* was overexpressed in HEI‐OC1 cells and cochlear explants to validate its role in enhancing autophagy and protecting HCs against D‐gal‐induced senescence. Post‐transfection with *Ronin*‐GFP plasmids and subsequent treatment with D‐gal for 72 h, western blot assay demonstrated a reduction in the levels of senescence‐related proteins (P16, γ‐H2A.X, and P21) compared with D‐gal treatment alone (**Figure** **7**A–F), suggesting that *Ronin* overexpression could mitigate cellular senescence in HEI‐OC1 cells. Furthermore, the reduction in γ‐H2A.X punctae in the nucleus confirmed that RONIN upregulation reduced HEI‐OC1 cellular senescence (Figure S5A, B Supporting Information). Additionally, enhanced autophagy was evidenced by increased levels of LC3B‐II, CTSB, and CTSD in *Ronin* overexpressing HEI‐OC1 cells (Figure 7G–L). Overexpression of *Ronin* in HEI‐OC1 cells resulted in an increase in LC3B‐II and p62/SQSTM1 levels but no significant changes were observed in the expression of Beclin1 and ATG7. Following treatment with bafilomycin A1, levels of LC3B‐II and p62/SQSTM1 were further accumulated, but there was no change in Beclin1 and ATG7 expression (Figure S5C–J). Autophagy flux was assessed through mCherry‐GFP‐LC3B lentiviral transfection in *Ronin*‐overexpressing HEI‐OC1 cells. The number of autophagosomes and autolysosomes significantly increased, and bafilomycin A1 treatment resulted in a greater accumulation of autophagosomes and autolysosomes compared to controls (Figure 7M, N). These results suggest that upregulation of *Ronin* expression activates autophagy and reduces D‐gal‐induced senescence in HEI‐OC1 cells.

**Figure 8:**
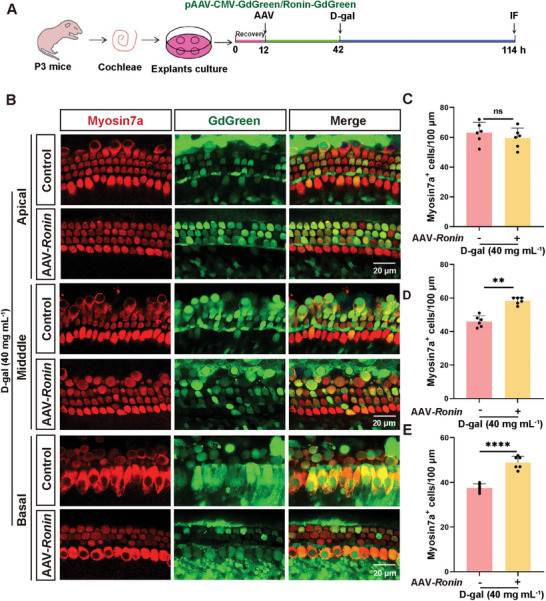
Protection against D‐gal‐induced HC loss in cochlear explants following overexpression of RONIN. A) Schematic diagram illustrating the experimental setup of *Ronin* overexpression in cochlear cultures in vitro, followed by treatment with D‐gal. AAV dose: 2 × 10^^^11 genome copies, D‐gal concentration: 40 mg mL^−1^. B) Immunofluorescence staining using Myosin7a antibody demonstrates loss of HCs expressing *Ronin*‐Gd Green in explants culture post‐D‐gal exposure. C–E) Quantification of Myosin7a‐positive HCs in the cochlea, n = 6. Error bars are ± S.D., ^**^ *p* < 0.01, ^****^ *p* < 0.0001, and ns: no significant difference.

Subsequently, the adeno‐associated virus 2.7m8 (AAV2.7m8),^[^ ^42^ ^]^ which can infect the cochlear inner HCs and outer HCs with high efficiency, was used to construct the AAV virus pAAV‐CMV‐*Ronin*‐3xFLAG‐EF1‐GdGreen‐WPRE (AAV2.7m8‐*Ronin‐GdGreen*). We utilized AAV2.7m8 to overexpress RONIN in cultured explants in vitro with over 75% transfection efficiency, as indicated by the GdGreen reporter (Figure S6, Supporting Information). Treatment of these infected cochlear explants with 40 mg mL^−1^ D‐gal for 72 h and subsequent staining with Myosin7a demonstrated a significant reduction in HC loss compared with treatment with D‐gal alone (**Figure** **8**A–E). Additionally, as indicated by SA‐β‐gal staining, the percentage of senescence‐positive HCs decreased significantly following RONIN overexpression (Figure S1C,D, Supporting Information). These findings indicate that *Ronin* overexpression not only promotes autophagy but also inhibits HC senescence, thereby enhancing the survival of senescent HCs.

## 3 Discussion

ARHL is the most common sensory impairment in older adults. This can result in several problems, including social isolation, frailty, and severe depression in later stages. These complications substantially influence the physical and emotional well‐being of older adults.^[^ ^43^ ^]^ Understanding the intricate causes and absence of specific therapies is vital to determining the fundamental processes of ARHL for successful interventions. The results of our study demonstrate a decrease in autophagy and a substantial increase in ROS levels in the D‐gal‐induced hearing loss model (Figure 4A–D), consistent with previous findings.^[^ ^20^, ^44^ ^]^ Additional investigation is required to clarify the intricate pathways that cause dysregulated autophagy.

Owing to the scarcity of cochlear tissue samples from aging deaf patients, which limits the study of age‐related deafness, senescent animal and tissue cell models have been widely utilized.^[^ ^45^ ^]^ In particular, the D‐gal‐induced senescence model, an aldohexose or reducing sugar model, is commonly used. At sufficient concentrations, D‐gal leads to cellular metabolic disorders, increased ROS levels, oxidative stress, inflammation, apoptosis, and senescence, making it ideal for constructing in vivo and in vitro senescence models.^[^ ^22^, ^46^ ^]^ We established a D‐gal‐induced senescence model in HEI‐OC1 cells and cochlear explants and also used naturally aged mice as depicted in Figures 1 and 2. We confirmed cell senescence by immunoblotting for aging markers, such as SMP30, P21, LaminB1, and γ‐H2A.X (Figures 1C–H and 2E–J, and L–O). Our findings, consistent with previous results,^[^ ^47^, ^48^, ^49^ ^]^ demonstrated increased ROS levels and accumulation of damaged mitochondria in D‐gal‐induced senescent HEI‐OC1 cells (Figure 4), indicating that mitochondrial dysfunction and mitophagy were repressed during the aging process. Mitochondrial dysfunction is implicated in the pathogenesis of chronic diseases and contributes to accelerated aging.^[^ ^50^, ^51^ ^]^ Consequently, we believe that promoting mitophagy holds significant potential for alleviating the burden of age‐related diseases.^[^ ^52^, ^53^ ^]^

TFEB serves as the principal regulator of autophagy and facilitates the translocation of autophagy‐regulated genes upon nuclear entry.^[^ ^54^ ^]^ Numerous studies have highlighted the significant effect of diminished nuclear localization of TFEB on neurodegenerative diseases and longevity.^[^ ^55^, ^56^ ^]^ Our findings revealed decreased total protein levels in aged HEI‐OC1 cells, cochlear explants, and mouse cochleae (Figure 5G–L). In the nucleus, TFEB can act as a transcription factor that activates the transcription and expression of autophagy‐related genes, such as p62 and Beclin1.^[^ ^57^ ^]^ The nuclear localization of TFEB increased significantly following treatment with 2 mg mL^−1^ D‐gal but decreased after treatment with 15 mg mL^−1^ D‐gal (Figure 5B, E). The marked increase of TFEB nuclear localization could explain the elevation in p62/SQSTM1 and Beclin1 protein levels in HEI‐OC1 cells after treatment with 2 mg mL^−1^ D‐gal (Figure 3B, E; Figure S2A, E, Supporting Information). Additionally, D‐gal treatment reduced *Tfeb* mRNA levels, suggesting inhibition of TFEB transcriptional activity (Figure 5M–O). Although the regulatory mechanisms of TFEB remain complex and are not fully understood, findings from the JASPER database and a dual luciferase reporter assay confirmed that RONIN enhanced TFEB transcriptional activity within a 1000 bp region upstream of the TFEB promoter (Figure 6A,K–M). In an in vitro ARHL model induced by D‐gal, upregulation of *Ronin* expression enhanced autophagy levels (Figure 7G–N; Figure S5C–J, Supporting Information), thereby ameliorating aging phenotypes (Figure 7A–F; Figures S1C, D and S5A, B, Supporting Information).

RONIN, a murine analog of human THAP11, has emerged as a novel pluripotency factor.^[^ ^58^ ^]^ RONIN regulates ribosomal gene transcription during development and is necessary for the self‐renewal of mouse embryonic stem cells.^[^ ^59^ ^]^ Additionally, it is pivotal in cardiac development by modulating key genes and enhancing mitochondrial gene activity in the developing retina, thereby contributing to mitochondrial function and cell cycle progression.^[^ ^36^, ^60^ ^]^ While recent studies have elucidated its broad biological functions, the molecular functions of RONIN in auditory system diseases remain largely unexplored. We inhibited the fusion of lysosomes and autophagosomes using bafilomycin A1 and measured the expression of p62/SQSTM1, Beclin1, LC3B‐II, and ATG7 after overexpressing *Ronin* in HEI‐OC1 cells and inducing aging with D‐gal. The results showed that in D‐gal‐induced senescent HEI‐OC1 cells, overexpression of *Ronin* promoted the expression of LC3B‐II and p62/SQSTM1 (Figure S5C, D, G, and H, Supporting Information). Regardless of whether bafilomycin A1 was used, overexpression of *Ronin* did not affect the expression of Beclin1 and ATG7 (Figure S5E, F, I, and J, Supporting Information). Overexpression of *Ronin* promoted LC3B‐II and p62/SQSTM1 expression, and treatment with bafilomycin A1 led to further accumulation of LC3B‐II and p62/SQSTM1. This is consistent with previous reports which show that overexpression of TFEB can activate the expression of LC3B‐II and p62/SQSTM1.^[^ ^57^ ^]^ These results validate the promoting effect of RONIN on autophagy. RONIN functions through the co‐activator HCF‐1, as it does not possess a distinct trans‐activation domain.^[^ ^41^ ^]^ We hypothesized that RONIN could bind to HCF1 to promote *Tfeb* transcription. Our results verified this hypothesis (Figure S4, Supporting Information). RONIN/HCF1 exerts physiological effects by activating the transcription of genes involved in protein synthesis and energy metabolism.^[^ ^41^ ^]^ TFEB facilitates lysosome production, whereas HCF‐1 is essential in cell cycle regulation.^[^ ^61^ ^]^ Therefore, RONIN/HCF1 may activate TFEB to protect against cochlear HCs senescence. In the future, RONIN/HCF1 should be modulated in vivo to prevent or delay aging‐induced HC injury.

Notably, existing research has demonstrated remarkably low expression of RONIN in adult animals.^[^ ^58^ ^]^ While we detected RONIN expression in the cochlear tissue of adult mice and reduced protein levels in aged mice compared with 1‐month‐old mice (Figure 6D, G). This discrepancy may stem from the samples originating from various organizations. Our findings indicate that RONIN likely plays a novel role in the mouse cochlea. Therefore, the mechanisms underlying RONIN's effects on autophagy in HCs merit further investigation. In addition, our study revealed significant findings; however, it is not without limitations. We only found that RONIN overexpression partially prevented D‐gal‐induced HC loss in vitro. In the future, we intend to investigate its protective effects in ARHL in vivo.

## 4 Conclusion

**Figure 9:**
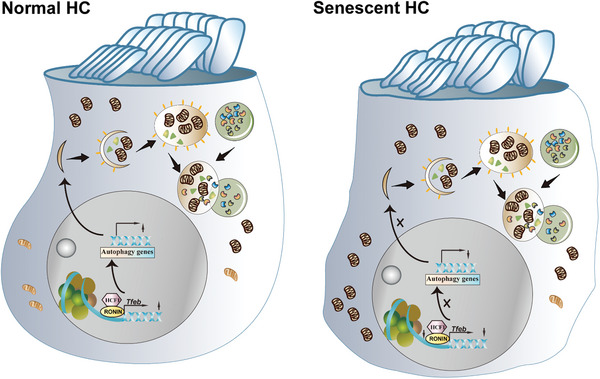
RONIN/HCF1 promotes *Tfeb* transcription to protect cochlea HCs from D‐gal‐induced damage. This schematic diagram presents the proposed mechanism by which the interaction between RONIN and HCF1 enhances *Tfeb* transcription, potentially preventing damage to cochlear HCs induced by D‐gal.

In summary, the findings of this study demonstrated that RONIN, in conjunction with HCF‐1, regulates the transcription of *Tfeb*. Overexpression of *Ronin* delayed senescence induced by D‐gal, revealing it as a promising target for clinical interventions aimed at treating natural aging‐induced hearing loss (**Figure** **9**).

## 5 Experimental Section

### Cell Culture

HEI‐OC1 cells were cultured in Dulbecco's modified Eagle medium (DMEM, Gibco, C11995500BT) supplemented with 10% fetal bovine serum (FBS, Pansera, P30‐2602) and 0.1% ampicillin (50 mg mL^−1^ ampicillin, Sigma‐Aldrich, A0166). The cells were maintained at a constant temperature of 37 °C and a CO_2_ concentration of 5%. Upon reaching 90% confluence, cells were trypsinized with 0.25% trypsin‐EDTA (Thermo Fisher Scientific, 25200054), following which these were seeded into either 6‐well plates (LABSELECT, 11110) or 24‐well plates (LABSELECT, 11310), as required. HEI‐OC1 cells were incubated overnight in 6‐well plates. After treatment with 0, 2, and 15 mg mL^−1^ D‐gal for 72 h, the cells were harvested. Following incubation with MitoSOX Red (2.5 µm) at 37 °C for 30 min, the cells were washed twice with PBS and suspended before being subjected to flow cytometry analysis (EX/EM, 510 nm/580 nm). MitoSOX‐positive cells were gated using the FlowJo software, and their relative proportion was quantified.

### Plasmids and siRNA Transfection

For transfection, HEI‐OC1 cells were cultured with plasmids using a transfection reagent from Invitrogen (11668027) in Opti‐MEM (Gibco, 31985062) following the manufacturer's instructions. pEGFP‐N3‐TFEB and RFP‐GFP‐LC3B plasmids were kindly provided by Zheng Ying (Soochow University), and the *Ronin*‐GFP plasmid was generated by Beijing Tsingke Biotech Company. The firefly luciferase reporter plasmid, pGL3‐*Tfeb*‐luciferase, and Renilla plasmids were synthesized by Beijing Tsingke Biotech. The promoter of the reporter was 1000 bp upstream of the transcription start site (TSS), as shown in Figure 6K. The siRNA sequences targeting *Hcf1* were synthesized by Shanghai GenePharma company: *Hcf1*‐Mus‐2996‐Forward: GUG CUC UGA UUU CCA AUC UTT, *Hcf1*‐Mus‐2996‐Reverse: AGA UUG GAA AUC AGA GCA CTT; *Hcf1*‐Mus‐3265‐Forward: GGC ACA ACU ACC AUU AUU ATT, *Hcf1*‐Mus‐3265‐Reverse: UAA UAA UGG UAG UUG UGC CTT; *Hcf1*‐Mus‐5955‐Forward: GCC CAU GGA UAC AUC UGA ATT, *Hcf1*‐Mus‐5955‐Reverse: UUC AGA UGU AUC CAU GGG CTT.

### Explant Culture and Virus Infection

Mice were obtained from The Core for Laboratory Animal Medicine, Institute of Health and Medicine, Hefei Comprehensive National Science Center, China. Cochlear explant cultures were prepared as previously described.^[^ ^62^ ^]^ In brief, P3 wild‐type FVB mouse cochleae were rapidly excised from the cochlear temporal bones. The cochlea was attached to the slides coated with Cell‐Tak (Corning, 354240), followed by the introduction of culture medium comprising DMEM, B‐27 (2%, Gibco, 17504044), N‐2 (1%, Gibco, A1370701), and 0.1% ampicillin. After recovery for 12 h, the explants were infected with AAV (pAAV‐CMV‐*Ronin*‐3xFLAG‐EF1‐GdGreen‐WPRE or control virus, 2 × 10^11 genome copies) for an additional 30 h before D‐gal treatment. All animal protocols were approved by The Core for Laboratory Animal Medicine, Institute of Health and Medicine, Hefei Comprehensive National Science Center (ethical approval number: IHM‐AP‐2024‐032).

### Drug Treatment

For cell culture experiments, 15 mg mL^−1^ D‐gal (in DMEM, Sigma‐Aldrich, G7050) was utilized to treat HEI‐OC1 cells for 72 h to construct an aging‐related cell model. For cochlear explant culture, after a 12 h recovery period, 20 and 40 mg mL^−1^ D‐gal were utilized to treat the explants.

### CCK‐8 Assay

HEI‐OC1 cells were treated with D‐gal for 72 h. Subsequently, 10 µL of the Cell Counting Kit (Protein Biotechnology, CC201‐01) reagent was introduced into each well to assess cell viability. The plates were incubated for an additional hour at 37 °C. The absorbance of the medium was measured at 450 nm using a microtiter plate reader (Bio‐Rad).

### Western Blot and Reagents

Cochleae from 12‐month‐old mice were ground after sacrifice by cervical vertebrae dislocation. Cochlear explants or HEI‐OC1 cells were harvested by low‐speed centrifugation. Samples were lysed using RIPA lysis buffer from Beyotime (P0013B) and a protease inhibitor cocktail (Roche, 04693132001), followed by high‐speed centrifugation after homogenization. For cytoplasmic and nuclear fractionation assays, HEI‐OC1 cells or mouse cochleae were harvested and separated using a Nuclear and Cytoplasmic Protein Extraction Kit (Beyotime, P0028) following the manufacturer's instructions. The BCA Enhanced Protein Assay Kit (Beyotime, P0010) was used to measure protein concentration. Protein samples were diluted in 5× SDS‐PAGE loading buffer (Hangzhou FUDE Biological Technology Co., Ltd., FD002). In brief, 20–30 µg protein was loaded in the 10–15% SDS‐PAGE gel (EpiZyme) in the running buffer after denaturation at 100 °C for 10 min. Separated proteins were transferred onto PVDF membranes (Millipore, IPVH00010) in the transfer buffer, and then the PVDF membranes were cut at a specific molecular weight and blocked in 5% skim milk (Beyotime, P0126) for 1–2 h at 20 °C. Subsequently, the primary antibodies were added and membranes were incubated overnight at 4 °C. The blot was incubated with secondary antibodies for 2 h at RT on the next day. Proteins were probed with the following primary antibodies, which served as loading controls: GAPDH (1:4000, Proteintech, 60004‐1‐Ig), ACTIN (1:40 000, Proteintech, 66009‐1‐Ig), and Tubulin (1:40 000, Proteintech, 11224‐1‐AP). Cell senescence was evaluated using anti‐Lamin B1 polyclonal (1:1000, Proteintech, 12987‐1‐AP), anti‐SMP30 (1:1000, Abcam, ab231322), anti‐phospho‐Histone H2A.X (Ser139) (γ‐H2A.X) (1:500, Cell Signaling Technology, 2577S), and anti‐P21 antibodies (1:1000, Abcam, ab109520). The mitochondrial mass was analyzed using anti‐COX4I1 and anti‐HSP60 antibodies (1:4000, Proteintech, 11242‐1‐AP, and 66041‐1‐Ig). Autophagy levels were assessed using anti‐LC3B, anti‐SQSTM1/p62, and anti‐Beclin1 antibodies (1:1000, Cell Signaling Technology, 2775, 23214, and 3495). and anti‐ATG7 monoclonal antibody (1:1000, Proteintech, 67341‐1‐Ig). Lysosomal function was determined with anti‐CTSD (1:400, Cell Signaling Technology, 2284) and anti‐CTSB (1:1000, Santa Cruz, sc‐365558) antibodies. Additional antibodies included anti‐THAP11/RONIN and anti‐TFEB (1:1000, Proteintech, 23030‐1‐AP and 13372‐1‐AP), anti‐HCF1/HCFC1 antibody (1:1000, Proteintech, 19358‐1‐AP and CST, 50708S). Goat anti‐mouse IgG HRP (1:5000, Abmart, M21001) and goat anti‐rabbit IgG HRP (1:5000, M21002) were secondary antibodies. Finally, SuperSignal West Dura Extended Duration Substrate from Thermo Fisher Scientific (34075) was used to detect proteins on the PVDF membranes. Protein bands were semi‐quantified using the ImageJ software.

### Immunofluorescence Assay

The samples (cells or cochleae) were fixed in 4% paraformaldehyde solution (Solarbio, P1110) at an ambient temperature for an hour and subsequently incubated in PBST (PBS [Bioshrp, BL302A] containing 1% Triton X‐100 [Solarbio, TB8200]) for 15 min. Samples were blocked by incubation with 10% donkey serum (Solarbio) for 1 h, followed by overnight incubation with primary antibodies at 4 °C. Cell aging was detected using the γ‐H2A.X antibody (1:100). The anti‐Myosin7a antibody (1:1000, Proteus Bioscience, 25–6790) was used as a marker for HCs. Mitochondrial ROS generation was examined by incubating cells with Mito‐SOX Red (1:1000, ThermoFisher Scientific, M36007) for 10 min at 37 °C. After incubation with the primary antibodies, the cells were incubated with the corresponding secondary antibodies, and nuclei were labeled with DAPI (1:1000, Solarbio, C0065). Following a second wash with PBST, the samples were mounted using an antifade mounting medium (DAKO, S3023). Confocal microscopy (Zeiss, Heidenheim, Germany, LSM780) was performed to capture detailed images of the cells and whole‐mount staining.

### Extraction of RNA and Real Time‐Quantitative PCR (RT‐qPCR)

Total RNA was extracted using the TRIzol Reagent (Life, 15596‐018), chloroform, and isopropanol, and synthesized into the corresponding cDNA using a cDNA synthesis kit (Thermo Fisher Scientific, K1622). The primer sequences used are mentioned in Table S1 (Supporting Information). RT‐qPCR was performed using the Bio‐Rad CFX96 Real‐Time PCR System (Bio‐Rad, Hercules, CA, USA). Previously described qPCR cycling conditions and gene expression culture methods were employed.^[^ ^63^ ^]^

### Measurement of Tfeb Promoter Activity

Cultured cells were inoculated in a 96‐well dish, and transfected with the *Tfeb* luciferase reporter plasmid, together with *Ronin*‐GFP, for 48 h. The Duo‐Lite Luciferase Assay Reagent (Vazyme, DD1205‐01) was used to measure firefly luciferase activity. Renilla luciferase activity was detected using Duo‐Lite Stop and Lite reagent. Both reactions were incubated for 10 min. The measurements were performed using a BioTek Cytation microplate reader. Renilla luciferase served as an internal control and was used to normalize the values.

### CUT & RUN‐qPCR Assay

The experiments were conducted according to the protocol specified in the Hyperactive pG‐MNase CUT & RUN Assay Kit for Illumina (Vazyme, HD102‐01). In brief, after exposing *Ronin*‐GFP expressing HEI‐OC1 cells to 15 mg mL^−1^ D‐gal for 72 h, the cell samples were collected in a solution of 0.25% trypsin‐EDTA, followed by resuspension in 100 µL of Wash Buffer. This suspension was combined with ConA Beads Pro, which contains a Binding Buffer. After subsequent centrifugation, the cell‐bead complex was resuspended in 100 µL of chilled Antibody Buffer, followed by the addition of the RONIN antibody (1:400, Thermofisher, MA5‐15674), and an overnight incubation at 4 °C. The complex was rinsed twice with 800 µL of Dig‐wash Buffer. Next, 100 µL of pre‐mixed pG‐MNase Enzyme was added, and the mixture was incubated in a rotor at 4 °C for 1 h. Following two additional washes, 100 µL of pre‐mixed CaCl_2_ buffer (from the CUT&RUN Assay Kit, HD102‐01, 2 µL CaCl_2_ was added to 98 µL Dig‐wash Buffer) was added, and the sample was incubated at a low temperature (ice) for 90 min. Subsequently, 100 µL of Stop Buffer was added before the sample was placed in a 37 °C water bath for 30 min. The supernatant was collected after centrifugation at 13400 x g for 5 min. DNA was subsequently extracted, and qPCR analysis was performed using the primer sequences synthesized for *Tfeb*: Forward‐CAC GGG CCA TCA TTG ACA G and reverse‐ GCC CGC TGG GAA ATG TAG T.

### Cellular and HC Senescence Detection

After HEI‐OC1 cells were treated with 15 mg mL^−1^ D‐gal, SA‐β‐gal staining was performed according to the protocol specified in the Cellular Senescence Detection Kit (SPiDER‐βGal, DOJINDO, SG03). To detect HC senescence after *Ronin* overexpression, SA‐β‐gal staining was performed using the senescence‐β galactosidase staining kit (Beyotime, China) following the manufacturer's protocol.

### Statistical Analysis

Data were analyzed using GraphPad Prism 9 software. Continuous variables were represented using average values and their respective standard deviations (Mean ± S.D.). Two‐tailed unpaired Student's t‐tests were used to determine statistical significance when comparing two groups. Differences between groups were assessed using one‐way ANOVA followed by Dunnett's post‐hoc test. Statistical significance was considered at a p‐value of less than 0.05 for all tests.

## Conflict of Interest

The authors declare no conflict of interest.

## Author Contributions

Y.‐j.W., Y.‐h.Z., W.C., and N.C. contributed equally as first authors to this work. Z.Y., R.‐j. C., Q.‐j.F., and J.‐m.Y. conceived and designed this project. Y.‐j.W., Y.‐h.Z., and N.C. executed most of the experiments and organized the figures. Y.X., L.G., and F.M. undertook the explants culture and image capture. Y.‐j.Z, Y.X., L.Z., J.S., B.‐w. S., and J.‐w.Y. helped with analyzing the data. Q.‐j.F., Y.‐h.Z., and W.C. wrote the original draft. S.‐h.S., and Z.Y. helped with approved this manuscript. Z‐h. H. helped revised the manuscript.

## Supporting information



Supporting Information

## Data Availability

The data that support the findings of this study are available in the supplementary material of this article.
