# Supplementary material for: RONIN/HCF1‐TFEB Axis Protects Against D‐Galactose‐Induced Cochlear Hair Cell Senescence Through Autophagy Activation
Source: Adv Sci (Weinh). 2025 Feb 22;12(29):2407880. doi: 10.1002/advs.202407880 (PMC12362728; doi:10.1002/advs.202407880)
Supplement: Supplementary file 1 — Supporting Information [file ADVS-12-2407880-s001.docx]

**Supporting Information**

**RONIN/HCF1-TFEB Axis Protects against D-galactose-Induced Cochlear Hair Cell Senescence through Autophagy Activation**

*Yongjie Wei, Yuhua Zhang, Wei Cao, Nan Cheng, Yun Xiao, Yongjun Zhu, Yan Xu, Lei Zhang, Lingna Guo, Jun Song, Su-hua Sha, Buwei Shao, Fang Ma, Jingwen Yang, Zheng Ying*, Zuhong He*, Renjie Chai*, Qiaojun Fang*, and Jianming Yang**

1. **Supplementary figures**

**
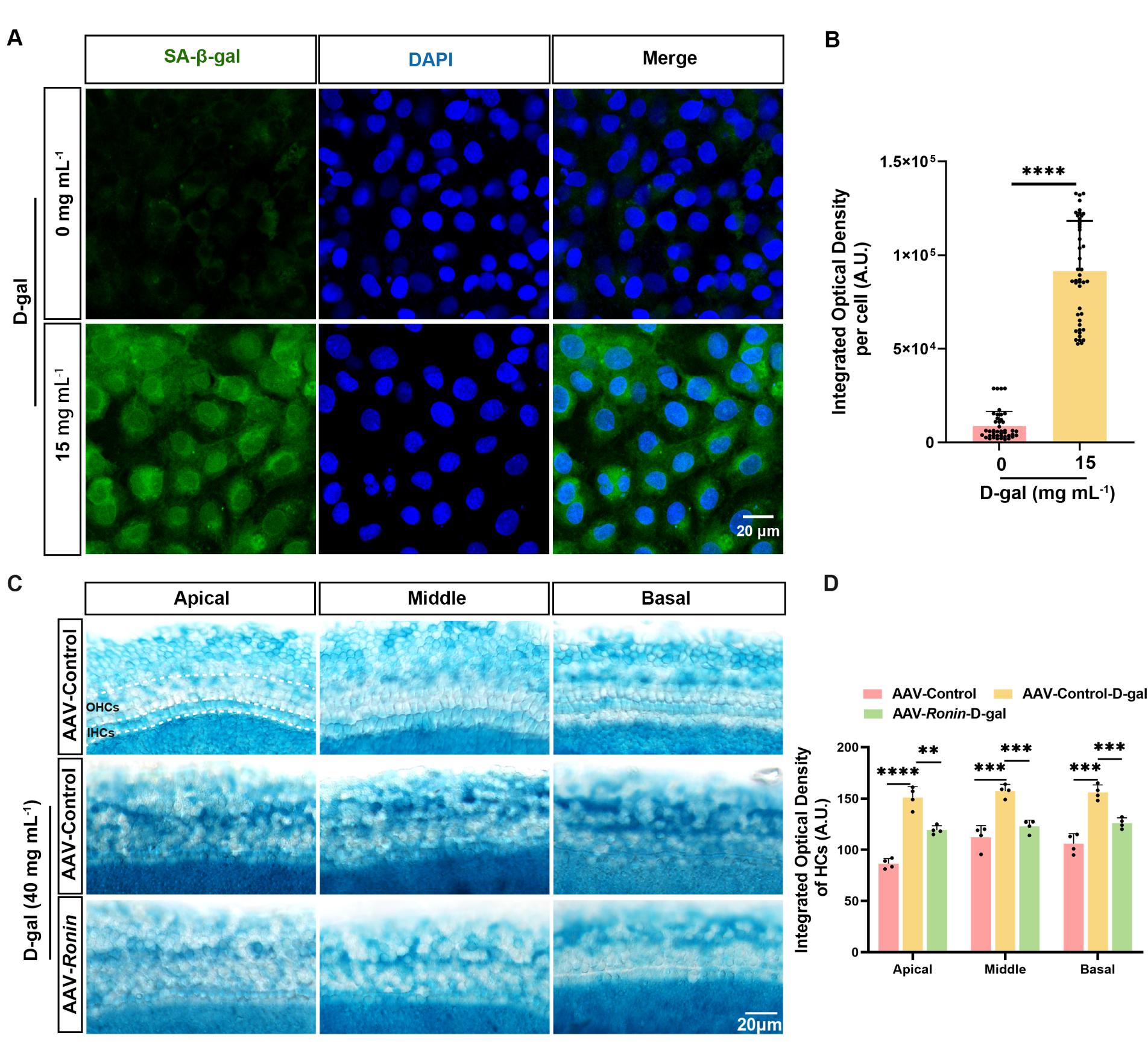
**

**Figure S1.** SA-β-Gal staining showed D-gal-induced senescence in HEI-OC1 cells and cochlear explants. A) Images of SA-β-gal staining (green) of HEI-OC1 cells after 15 mg mL^-1^ D-gal treatment for 72 h, Scale bar: 20 μm. B) Analysis of integrated optical density of SA-β-gal (green) per HEI-OC1 cell. n = 45. Error bars are ± S.D., ****p<0.0001. C) SA-β-gal staining images of HCs in the middle turns of cochleae after D-gal treatment. Cochlear explants infected with AAV for 30 h followed by treatment with 40 mg mL^-1^ D-gal for 72 h, Scale bar: 20 μm. D) Quantification of integrated optical density in HCs. n = 4. Error bars are ± S.D., **p<0.01, ***p<0.001. and ****p<0.0001.

**
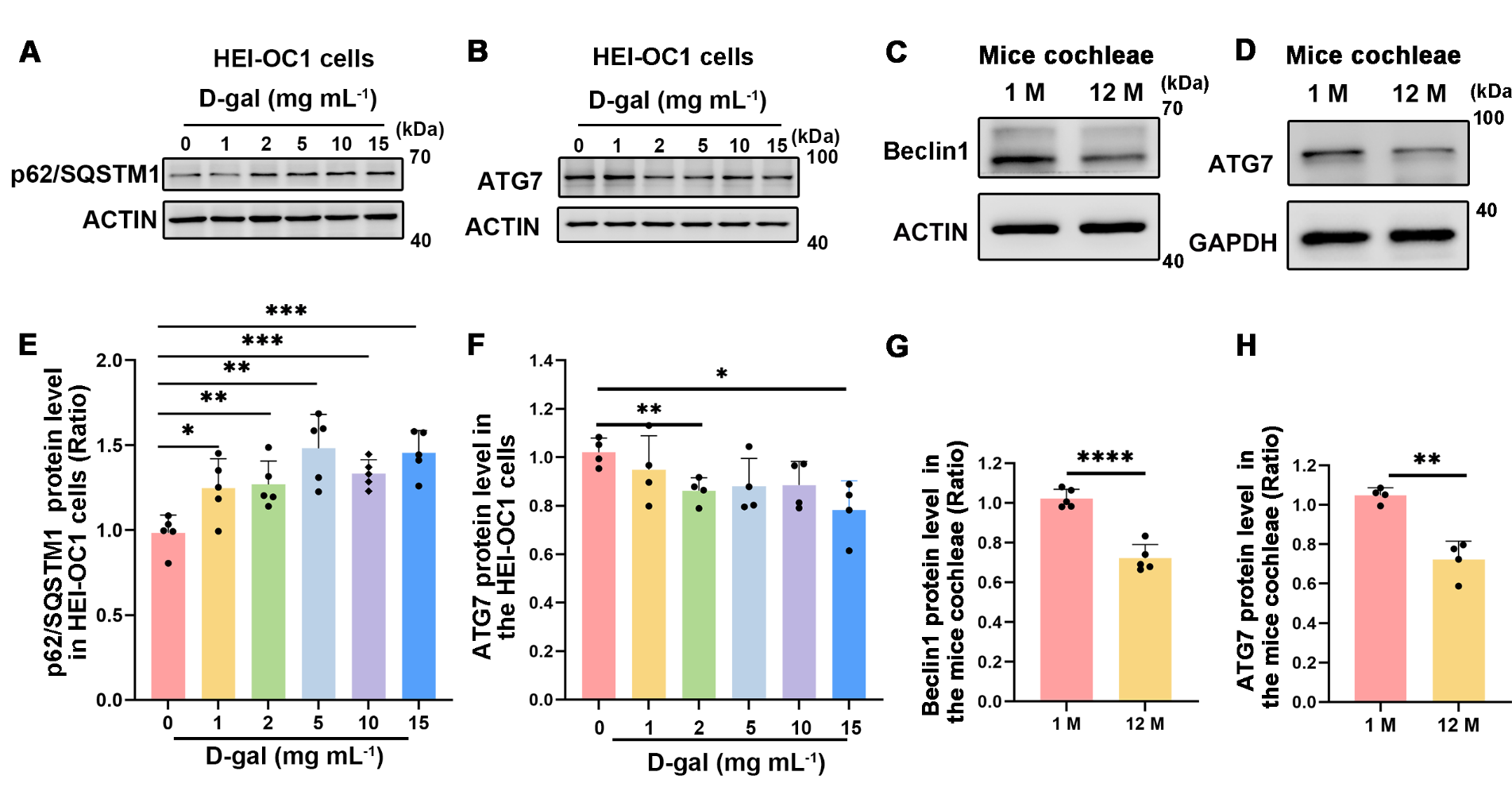
**

**Figure S2.** Autophagy-related marker expression in aging HEI-OC1 cells reduced following D-gal treatment and in naturally aging mice. A-B) Western blot analysis demonstrates alterations in p62/SQSMT1 and ATG7 protein levels in HEI-OC1 cells following treatment with different concentrations of D-gal for 72 h. C-D) The western blot shows Beclin1 and ATG7 protein levels in natural aging mice. E) Quantification of the western blot results in A, n = 5. Error bars are ± S.D., *p<0.5, **p<0.01 and ***P<0.001. F) Quantification of the western blot results in B, n = 4. Error bars are ± S.D., *p<0.5, **p<0.01. G) Quantification of western blot results in C, n = 5. Error bars are ± S.D., ****p<0.0001. H) Quantification of western blot results in D, n = 4. Error bars are ± S.D., **p<0.01.


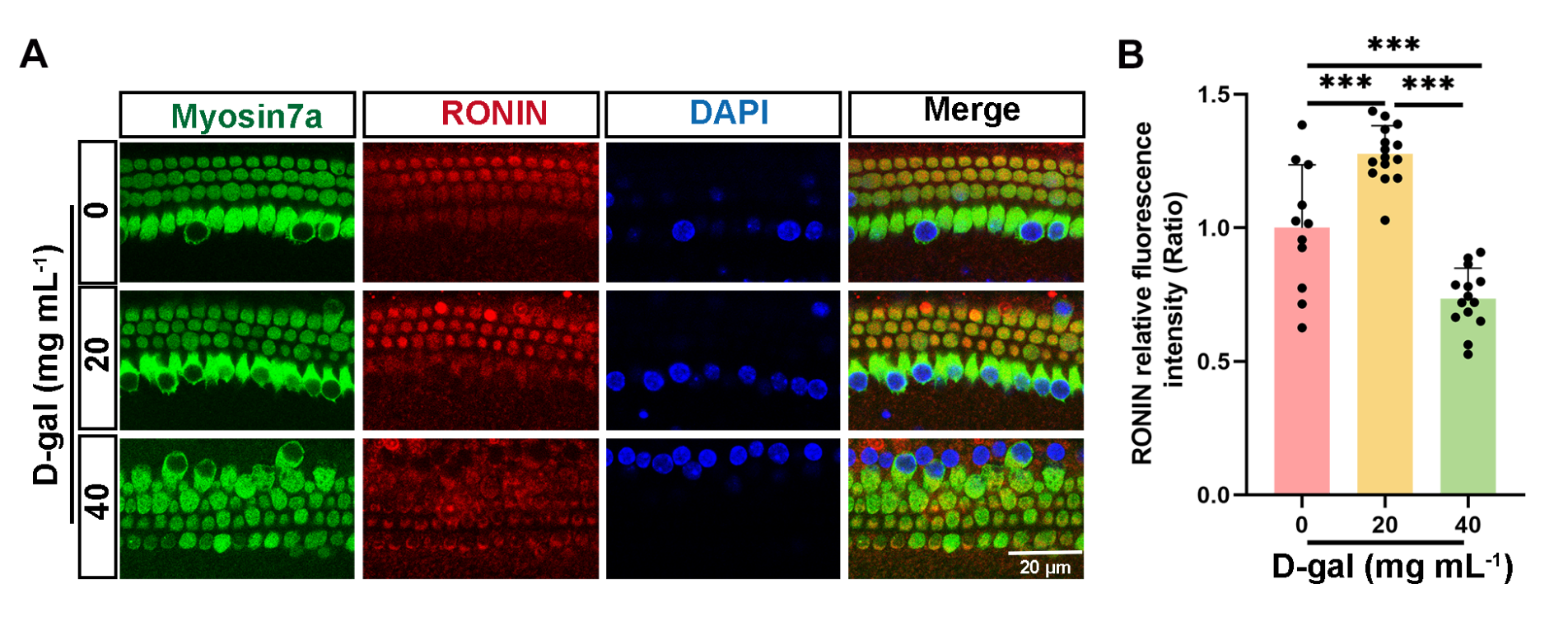


**Figure S3.** RONIN expression decreased in cochlear explants after D-gal treatment for 72 h. A) immunofluorescence staining for Myosin7a and RONIN shows the expression of RONIN in cochlear HCs after D-gal treatment for 72 h, Scale bar: 20 μm. B) Quantification of fluorescence intensity of RONIN, n = 11. Error bars are ± S.D., ***p < 0.001.


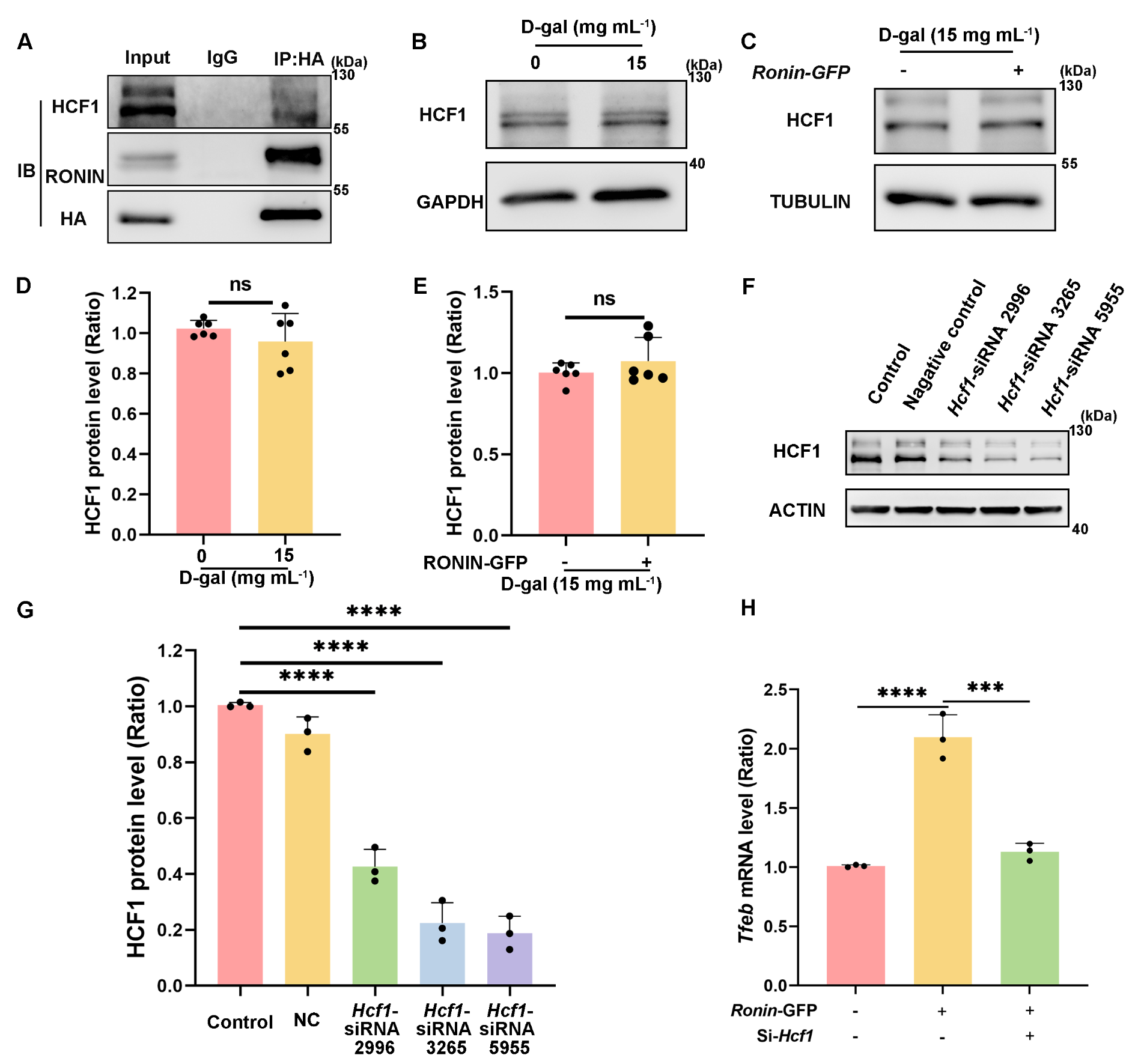


**Figure S4.** RONIN interacts with HCF1 to promote *Tfeb* transcription. A) Immunoprecipitation assay was used to detect the interaction of RONIN and HCF1. HEI-OC1 cells were cultured for 48 h after transfection with *Ronin*-HA. B) Western blots show HCF1 protein levels after 15mg mL^-1^ D-gal treatment for 72 h. C) Western blots show changes in HCF1 protein levels after transfection with *Ronin*-GFP for 48 h and D-gal treatment. D-E) Quantification of HCF1 protein level, n = 6. Error bars are ± S.D., ns: no significant difference. F) HEI-OC1 cells were transfected with four different siRNAs targeting mouse *Hcf1,* and *Hcf1* siRNA 5955 showed the most effective knockdown of HCF1. G) Quantification of western blots results for HCF1, n = 3. Error bars are ± S.D., ****p<0.0001. NC: negative control. H) qPCR indicating *Tfeb* mRNA levels. HEI-OC1 cells were transfected with *Hcf1* siRNA 5955 for 24 h, and the cells were re‐transfected with *Ronin*-GFP for another 24 h. n = 3. Error bars are ± S.D., *** p < 0.001 and **** p < 0.0001.

**
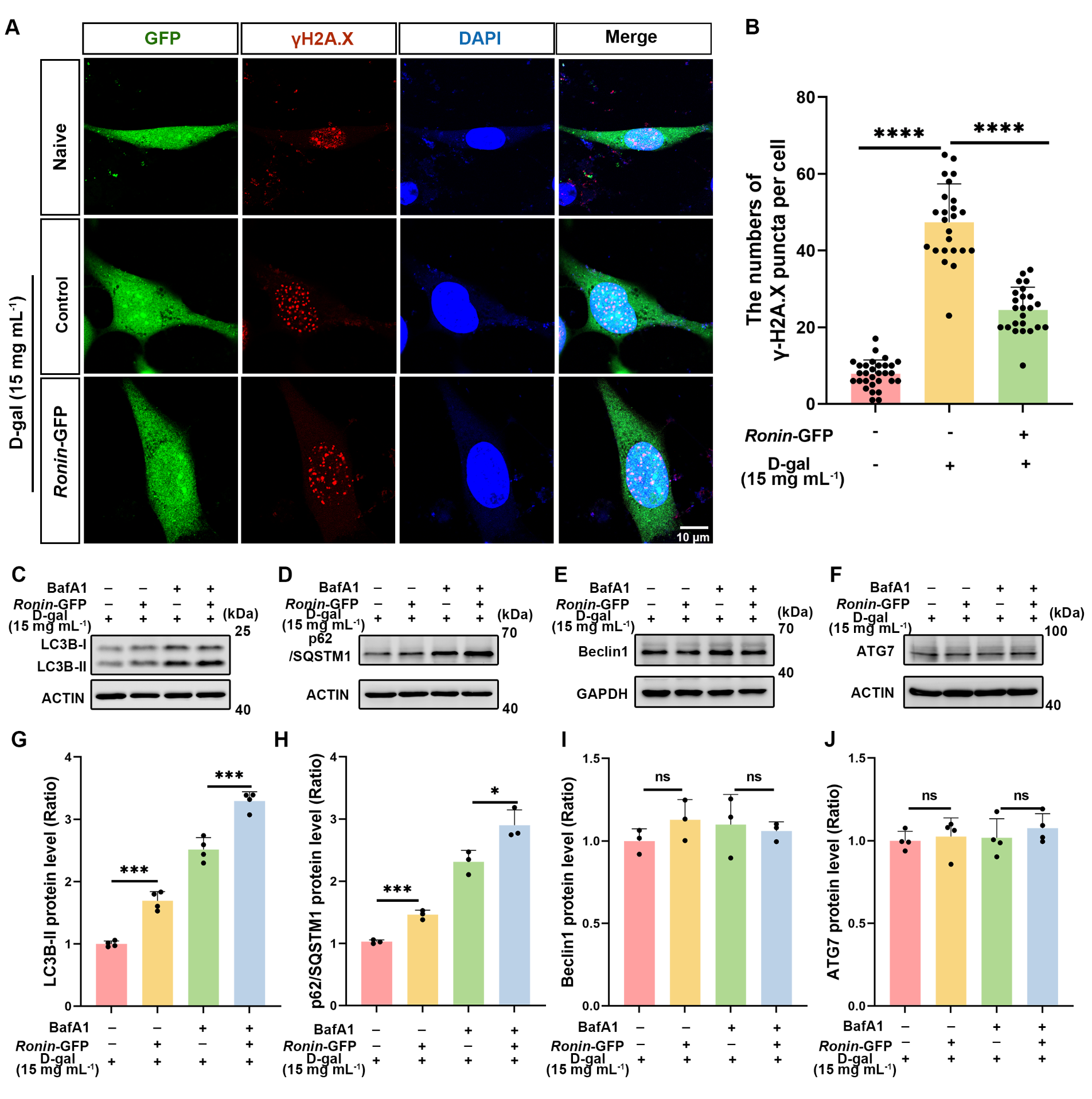
**

**Figure S5.** The number of γ-H2AX foci decreased and autophagy level increased in HEI-OC1 cells expressing *Ronin*-GFP. A) Immunofluorescence imaging of γ-H2AX foci in HEI-OC1 cells expressing *Ronin*-GFP after treatment with 15 mg mL^-1^ D-gal for 72 h. Naïve group: HEI-OC1 cells were only treated with control plasmid. B) Quantitative analysis of γ-H2A.X foci in the nuclei, n = 24. Error bars are ± S.D., ****p<0.0001. C-F) Western blot examination shows alterations in the protein levels of LC3B-II, p62/SQSTM1, Beclin1, and ATG7 in HEI-OC1 cells. Cells were transfected with *Ronin*-GFP plasmids, followed by exposure to 15 mg mL^-1^ D-gal for 72 h with or without 100 nM BafliomycinA1 (BafA1) for 12 h. G) Quantification of the western blot bands of LC3B-II in C, n = 4. Error bars are ± S.D., ***p<0.001. H) Quantification of p62/SQSTM1 protein levels in D, n= 3. Error bars are ± S.D., *p<0.05 and ***p<0.001. I) Quantification of the western blot bands of Beclin1 in E, n = 3. Error bars are ± S.D., ns: no significant difference. J) Quantification of the western blot bands of ATG7 in F, n = 4. Error bars are ± S.D., ns: no significant difference.


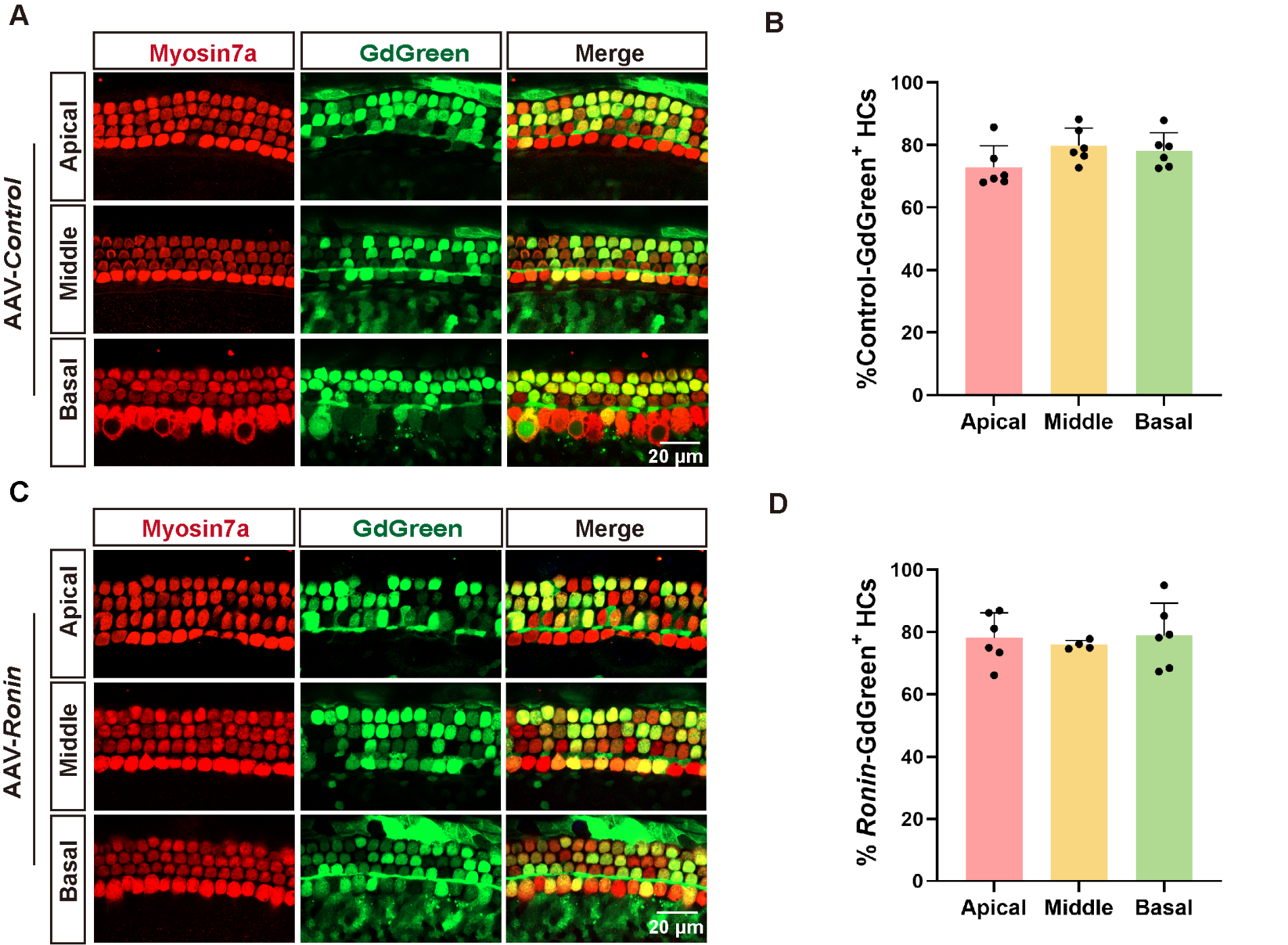


**Figure S6.** Infection efficiency of AAV-control and AAV-*Ronin*. A and C) Representative images of cytoplasmic colocalization of Control/*Ronin*-GdGreen fluorescence (green) and Myosin7a (red) in apical, middle, and basal turns of cochleae after 48 h of AAV infection. B and D) Percentage of GdGreen-positive HCs per 100 µm, n = 6. Error bars are ± S.D.,

1. **Supplementary table**

**Table S1. qPCR Primer sequences**

| **Gene Name** | **Direction** | **Sequences** |
| --- | --- | --- |
| *Ronin* | Forward | GGT GGG TTG GAA GGT GGA G |
|  | Reverse | ATG CCT GGC TTT ACG TGC T |
| *Tfeb* | Forward | CCA GAA GCG AGA GCT CAC AGA T |
|  | Reverse | TGT GAT TGT CTT TCT TCT GCC G |
| *Hcf1* | Forward | CGG CAA CGA GGG GAT AGT G |
|  | Reverse | TAG GCG AGT ACC ATC ACA CAC |
| *GAPDH* | Forward | AGG TCG GTG TGA ACG GAT TTG |
|  | Reverse | TGT AGA CCA TGT AGT TGA GGT CA |
